# Shared decision‐making in standardized cancer patient pathways in Norway—Narratives of patient experiences

**DOI:** 10.1111/hex.13317

**Published:** 2021-07-21

**Authors:** Tone Andersen‐Hollekim, Line Melby, Kari Sand, Heidi Gilstad, Anita Das, Marit Solbjør

**Affiliations:** ^1^ Department of Public Health and Nursing, Faculty of Medicine and Health Sciences NTNU Trondheim Norway; ^2^ Department of Health Research, SINTEF Digital SINTEF Trondheim Norway; ^3^ Centre for Academic and Professional Communication, Department of Language and Literature NTNU Trondheim Norway

**Keywords:** cancer, cancer patient pathway, narrative, Norway, patient participation, shared decision‐making, standardization

## Abstract

**Background:**

Cancer patient pathways (CPPs) were implemented in Norway in 2015–2017 to advance cancer diagnostics and treatment initiation. The aim of CPPs is to ensure standardized waiting times, but also to strengthen patient participation and shared decision‐making. This study investigates how patients enrolled in a CPP experienced shared decision‐making.

**Methods:**

This study comprised of 19 individual semistructured interviews with patients who had been enrolled in a CPP at three hospitals in Norway. Twelve patients had breast cancer, four patients had prostate cancer and three patients had malignant melanoma. We analyzed their experiences using a narrative approach.

**Findings:**

This study showed how participating in a standardized CPP provided different possibilities for shared decision‐making. The patients' narratives of shared decision‐making in CPPs included stories from the three cancer diagnoses through the following themes: (1) The predictable safeness of standardizations, (2) the ambivalence of making decisions and (3) opposing standardizations and pushing for action.

**Conclusion:**

Standardized CPPs provided patients with predictability and safety. Shared decision‐making was possible when the cancer diagnoses supported preference‐sensitive treatment options. Balancing standardizations with individualized care is necessary to facilitate patient participation in CPPs, and the possibility of shared decision‐making needs to be discussed for each specific CPP.

**Patient or Public Contribution:**

A service user representative from the Norwegian Cancer Society participated in designing this study.

## 1 INTRODUCTION

Standardized cancer patient pathways (CPPs) have been implemented country by country in Scandinavia, beginning with Denmark in 2007.^1^ CPP initiation was based on an unwanted variance in cancer treatment, especially in terms of waiting times and treatment options across hospitals. In Norway, CPPs were launched in 2015.^2^ Their purpose is to ensure that cancer patients experience a well‐organized, comprehensive and predictable trajectory without unnecessary non‐medically justified delays in assessment, diagnostics and treatment, including a strong emphasis on patient participation. A CPP is typically initiated by a general practitioner (GP), who, upon reasonable cancer suspicion, sends a referral to the hospital for further investigation by the specialist health service. The CPP commences when the specialist health service receives the referral, after which the health service should respond within the time frame defined for each phase in each CPP. As an example, in one of the 28 CPPs that have been implemented in Norway, the breast cancer pathway is divided into three phases. It comprises 14 days for the first two phases. These phases include the periods from receipt of referral by the hospital until the first appointment for procedures and tests in the pathway and from the first appointment for procedures and tests until the assessment is completed. The third phase is the period from being diagnosed as having cancer until the start of treatment, which should comprise no more than 13 days.^3^

Standardization of CPPs is meant to ensure predictability, accountability and objectivity for patients and health services^1^ and is an important part of modern healthcare.^4^ Standardization does, however, carry a risk of dehumanization if patients are seen as objects that should fit into a standard.^5^ Over the last few decennials, health services have been subject to democratization processes meant to counteract objectivation of patients through the initiation of patient participation, which is included as a policy aim for the Norwegian CPPs.^2^ Policy documents on CPPs state that patients and next of kin should receive individually customized information, be involved and participate in dialogue and that decisions should be made jointly between patients and physicians.^6^ The focus on patient participation in the Norwegian CPPs could, however, create a paradox as standardization and individualization are two seemingly opposing trends. Norwegian political speeches during the initiation of the CPPs solved this by constructing good patient treatment as ‘individualized standardization’.^4^ Such constructions could potentially allow for negotiations of standards if patients are involved in service design or through participating in shared decision‐making with health professionals. However, little is known about how patients experience patient participation within a standardized cancer pathway. The present article explores patient participation within standardized CPPs during the phases of examinations, diagnostics and treatment decisions.

Patient participation is advocated as an important part of modern medicine, including cancer care.^7^, ^8^ Shared decision‐making is one of several approaches to achieving patient participation and is especially suitable when more than one treatment option exists and the options are considered equal with regard to outcome.^9^ Shared decision‐making is a process in which healthcare professionals and patients work together to make decisions about treatment and management based on clinical evidence and patient preferences.^9^ In shared decision‐making, the healthcare professional informs the patient, explains the pros and cons of each option, discusses the patient's preferences with the patient, including how the patient prefers to make decisions and finally makes or defers a decision.^10^ Sharing the decision could also be relevant for decisions related to logistics or other practical issues.^8^ Thus, the nature of decision‐making in the different encounters will vary according to the purpose of each encounter.

A cancer diagnosis confronts patients profoundly with their mortality,^8^ and insecurity and vulnerability may follow.^11^ Healthcare professionals could underestimate patients' ambivalence and reduced decision‐making capacity when faced with the unfamiliar field of medicine.^11^ The degree to which patients prefer to participate in decision‐making about treatments varies between types of cancers and the characteristics of the patients,^12^ but most patients prefer an active role in decision‐making.^8^, ^13^ However, patients are found to be more confident about making decisions that do not require medical knowledge and often prefer their physicians to provide a treatment direction or recommendation.^9^, ^14^, ^15^ Some clinicians are reluctant or ambivalent when recommending a preference‐sensitive treatment, out of respect for patient autonomy.^15^, ^16^ Patients' need to participate may differ across the cancer trajectory, and patients often become more confident in decision‐making as their trajectory unfolds.^8^ Paradoxically, key decisions are made in the initial stages, when relationships are new and emotionality is intense.^8^

Although widely advocated, shared decision‐making exists in varying degrees in clinical practice. Barriers are related to patients and healthcare professionals as well as the healthcare organization.^16^, ^17^ According to traditional healthcare practice, clinicians are, by the nature of their profession, expected to make decisions, and patients are expected to follow medical recommendations.^18^ Patients may feel reluctant or anxious about sharing decisions^16^ and depend on professional expertise to restore their health.^11^ This may lead them to apply a ‘doctor knows best’ attitude and defer from participating.^16^, ^19^ Avoiding disagreement with medical recommendations may be a way for patients to ensure a healthy relationship with care providers.^19^, ^20^

Previous research suggests deficiencies in shared decision‐making in cancer care. For instance, a Danish study found that breast cancer patients had unmet needs related to information, communication and involvement in treatment choices, even when the CPP was experienced in a fast and well‐organized manner.^21^ A German study found that even though shared decision‐making lacked implementation in German breast care centres, nurse‐led decision coaching based on evidence‐based information increased shared decision‐making.^22^ Similar issues exist for prostate cancer, where a study analyzing educational material concluded that the material was overly complex and not readable for laypeople.^23^ When deciding on treatment options, not all prostate cancer patients were offered a choice by their clinicians.^7^ Another study found that treatment decisions were considered to be part of the physician's role as both healthcare professionals and patients doubted the latter's abilities to participate in decisions.^24^ Therefore, treatment information was emphasized and amounted to patient involvement.^24^ Studies on shared decision‐making for malignant melanoma trajectories are scarce, although it has been found that most melanoma patients prefer an active role and that implementing a shared decision‐making approach in clinics increased patient involvement.^25^ With the implementation of standardized patient pathways in cancer care across countries, more knowledge is needed about how patients experience shared decision‐making in a standardized CPP.

### 1.1 Aim of this study

The aim of this study was to explore the experience of patients who had undergone standardized CPPs, specifically their involvement in shared decision‐making through their CPP.

## 2 METHODS

### 2.1 Design

This was a retrospective, qualitative interview study with individuals who had been patients in a standardized CPP in Norway. The study was part of a larger research project on CPPs in Norway. CPPs that represented both common and rarer forms of cancer were included. Although the main project also included lung cancer, this study omitted this CPP due to ethical reflections on lung cancer patients' potentially poor health at the end of the CPP. The present study thus included three diagnostic groups: breast cancer, prostate cancer and malignant melanoma. This selection allowed exploration of variations in patient experiences due to the different characteristics of these cancers and CPPs.

### 2.2 Setting

Norwegian healthcare services are built on universal health insurance. There are four regional health trusts in Norway, including both local and university hospitals, which attend to patients in need of specialized health services such as cancer surgery. Cancer patients who are initially examined at a local hospital are most often transferred to the university hospital for specialized examinations and treatment. However, chemotherapy could be administered by a local hospital. Preoperative assessments, comorbidity and communication with each patient form the basis for individualized trajectories. Patients are primarily treated within the geographical region where they live, but the right to choose between hospitals is regulated by law.^6^ Cancer patients receive individually adapted evidence‐based treatment. For women with breast cancer, the choice of treatment includes breast‐conserving surgery or mastectomy, chemotherapy and/or radiation therapy.^6^ Depending on the stage of cancer, treatment options for men with prostate cancer include active monitoring, curative treatment, such as radical prostatectomy or radiation therapy with or without endocrine treatment and symptom‐oriented treatment, possibly including endocrine and/or palliative care.^6^ In patients with malignant melanoma, treatment relates to primary or extended excision, lymph node dissection, surgical metastasis removal and/or oncological treatment.^6^

### 2.3 Recruitment and sample

The participants were recruited through three university hospitals within three of the four regional health trusts in Norway, respectively, in the northern, central and southern parts of the country. One of these hospitals serves the region with the highest population density, whereas another serves the region with the lowest population density and longest travel distances. The northern hospital recruited patients with breast cancer and malignant melanoma, the central hospital recruited patients with breast cancer, prostate cancer and malignant melanoma and the southern hospital recruited patients with prostate cancer. All patients who had been in a CPP during the last few months were eligible for inclusion. A nurse in each hospital department identified patients, beginning recruitment with those patients who had recently finished their CPP and counting backwards. Each hospital department was asked to recruit up to 15 participants, based upon the available number of patients and expectations regarding data saturation.

Breast cancer patients received information about the study from a nurse during an appointment at the clinic, as did prostate cancer patients at the central hospital. All patients with malignant melanoma and men with prostate cancer at the southern hospital received study information by ordinary mail and had to phone a researcher to be included in the study. One participant self‐recruited after hearing about the study in an informal setting.

A total of 19 participants took part in the project. Five were men, four of whom had prostate cancer and one had malignant melanoma. Two women had malignant melanoma, whereas 12 had breast cancer (Table 1). All patients had completed a CPP, which meant that they had commenced treatment. Some of the women with breast cancer were still receiving either chemotherapy or radiation at the time of the interview, whereas all other participants had finished treatment.

**Table 1:** Participant characteristics

Cancer patient pathway	Participants (*n*)	Age span (years)	Mean age (years)
Breast	12	40‒64	53
Prostate	4	64‒75	71
Melanoma	3	50‒69	62
All patients	19	40‒75	58

### 2.4 Data collection

Individual interviews were conducted by three researchers who all had substantial experience with qualitative interviewing. Seven interviews were performed by phone due to long travel distances, while the remaining 12 interviews were conducted face to face. One patient was interviewed at home, one in a meeting room in a public building near the patient's home and the remaining 10 interviews were carried out in a meeting room either at the university or at the hospital. The phone interviews lasted from 20 to 64 min (mean 34), whereas face‐to‐face interviews lasted from 31 to 75 min (mean 49). We used a semistructured interview guide, which was developed by the research group (the development of the interview guide was led by Line Melby and Marit Solbjør) to explore patients' experiences of CPPs. It covered the main aspects of the policy aims for the Norwegian CPPs: How the patient's CPP was initiated, waiting time, information, patient participation and shared decision‐making (Table 2). The interview guide was adapted to each interview situation, allowing patients to focus on what they had found most important during their cancer trajectory. During each interview, participants were encouraged to narrate their experiences with CPPs and spoke freely about being diagnosed with cancer and going through treatment. With regard to saturation, this was discussed during data collection. We considered the data from the breast cancer pathway saturated after 12 interviews. The process of recruiting patients from the prostate and melanoma CPPs proved more difficult and recruitment ended due to a lack of patients who fulfilled the inclusion criteria at each hospital.

**Table 2:** Interview guide and questions on patient participation

Themes	Questions
Intro	Diagnosis, hospital, timeline for CPP
Concept	What do you understand by the term ‘CPP’?
	Have you heard about CPP?
Beginning the cancer patient pathway (CPP)	Please tell us about how you got your cancer diagnosis.
	–Who was your first contact?
	–How did the GP meet you?
	What information about CPP did you receive at the GP?
	–Did you search for information on CPP?
Waiting time between first contact with general practitioner (GP) and start of treatment	How did you experience the time between your first contact with the GP and your first contact with the hospital?
	What information did you receive about your future examinations and treatment?
At the hospital	Who was your contact at the hospital, and how did they meet you?
	What information about CPP did you receive from the hospital?
	Have you experienced being in a CPP?
	How did you experience the time frame from your first encounter with the GP until your treatment start‐up?
Information through the CPP	Have you been informed about the procedures during your cancer patient pathway?
	Did you miss any information?
Patient participation	What decisions were made at the GP's?
	–Who made these decisions?
	How did you participate in decision‐making during your cancer pathway?
	–Did you have sufficient information to take part in decision‐making?
	Did you experience decision‐making in which you did not take part?
	–How would you have preferred to participate in decision‐making?
	Have you ever wanted to take part in decision‐making without being heard?

### 2.5 Data analysis

Although the interviews were based on a semistructured interview guide, participants were eager to tell their cancer stories, including stories about CPP and decision‐making. This provided thick data on each step of the CPP and subsequently led us to choose a narrative approach to the analyses. A narrative analysis reflects an individual's told experience of an event, related as a story with a beginning, a middle and an end, with causal incidents organized in a plot.^26^ To analyze the data, all authors read some of the interviews and contributed to discussions on what were the main themes within the stories throughout the analytical process. Tone Andersen‐Hollekim and Marit Solbjør read all the transcribed interviews several times and conducted the coding of each interview. The first reading allowed an overall impression of the text. In the following reading, we identified codes by choosing words or short phrases in the text.^27^ Codes with similar meanings were grouped together, which enabled us to observe patterns in the material. We then organized the codes into categories according to these patterns. In accordance with a narrative approach,^26^ we searched for similarities and contradictions, turning points or shifts in the patient stories. The analysis was conducted using an iterative process, in which we continuously rechecked the developing themes with the transcripts.

### 2.6 Ethics

The study was approved by the Norwegian Centre for Research Data. All study participants provided written informed consent. Participation was voluntary and all patients invited to the study were informed that their treatment or health services would not be influenced by their participation or nonparticipation in this study.

## 3 FINDINGS

Our findings show how patients with breast cancer, prostate cancer and malignant melanoma experienced shared decision‐making in their standardized CPP. The findings elucidate the tensions between standardization and individualization in healthcare, as illustrated in the narratives' overall plotlines. The narrative approach shows how individuals adapt to medical standardizations while still desiring individualized care. Most patients in our study were unfamiliar with the term ‘CPP’ and could not recall that healthcare providers had mentioned this term during their trajectory. However, they found that medical examinations and assessments were organized in a logical order, which they associated with a standardized patient pathway. Although diagnostics and treatment choices in all three CPPs followed evidence‐based protocols, the breast and prostate CPPs additionally featured preference‐sensitive medical decisions. For instance, women with breast cancer had to decide whether to undergo breast‐conserving surgery, whereas men with prostate cancer had to decide on active monitoring or immediate radical treatment. Patients who had malignant melanoma did not take part in any form of decision‐making.

In the following section, we present the patients' narratives of shared decision‐making in CPPs as one narrative, including stories from the three cancer diagnoses through the following themes: (1) the predictable safeness of standardizations, (2) the ambivalence of making decisions and (3) opposing standardizations and pushing for action.

### 3.1 The predictable safeness of standardizations

Women diagnosed with breast cancer described two routes into the CPP. Some experienced detecting a lump in their breast, after which they contacted their GP and were referred to specialist healthcare. Others had taken part in a mammography screening programme, from which they were directly enrolled in the CPP. Receiving the cancer diagnosis became a significant turning point in the life of these women, representing a transition into illness. Becoming a patient suffering from a potentially life‐threatening disease led to uncertainty, with a plethora of questions arising. In the stories of these women, the CPP and the standardization of treatment options that they were presented with provided predictability and safety at a time when their lives were disrupted and threatened. Although standardization represented security, it was still important for the women that healthcare professionals recognized them as individuals. This became visible in the way they sought information beyond standardization, looking for information tailored to each unique situation: ‘The information you get is quite general, not very in‐depth…. I would like to have had more information about my type [of cancer]’. (Participant 1)

The women told of receiving valuable information about their diagnosis and treatment trajectory. Nevertheless, their experience was that the CPP and treatment decisions were conducted based on evidence‐based knowledge, and therefore did not require patient involvement in the decision‐making process. Typically, in their stories, healthcare providers told them how treatment would proceed. The standardized pathway helped women to solve questions of uncertainty that arose in the initial phase of the cancer diagnosis. As one of them stated, ‘It feels like someone has figured out a clever way to do this, and it is thus reasonable for me to follow this way’ (Participant 2). Trust in the health system and in professional competence pervaded their stories, allowing them to take a passive position in decision‐making through actively leaning on professional competence.

Even though the standardized breast cancer pathway provided security, it allowed less space for shared decisions. As one of the women pointed out, ‘I found them [health personnel] to be very considerate, caring, and emphatic, but I can't say that I experienced participating in decision‐making’ (Participant 11). Not all women felt comfortable with agreeing to decisions made on their behalf and recalled being sent back and forth in the healthcare system without having a say. Although treatment was voluntary, they found that healthcare professionals expected them to submit to standardized treatment recommendations. Patients' decision‐making became limited to logistics, such as deciding on time schedules.

The patients with malignant melanoma told of detecting suspicious moles on their bodies after which they scheduled appointments with their GP. Patients were enrolled in the CPP when the malignancy suspicion was confirmed, either by biopsy or by visual examination. These participants talked about having been through a fully standardized pathway without experiencing any preference‐sensitive medical treatment options. In their narratives, surgery was the option, and healthcare professionals instructed them on when and how it should be carried out. Putting significant trust in healthcare professionals, this option represented a safe solution for the patients, as stated by these participants:

‘It was just like, “This is how it is; this is what we do”. No problem, I was taken care of, I leave it to them. They know best, and I don't have any objections’. (Participant 18).

‘I have not taken part in any decisions, I reckon they know their stuff […] When you are under the care of health personnel, you will be notified if there is anything’. (Participant 19).

Not presented with different choices, the melanoma patients did not seek to make decisions. Rather, they positioned themselves as passive care recipients and were led safely along the pathway by healthcare professionals. However, although patients preferred this role in treatment decisions, one of them suggested that if treatment could not be combined with everyday plans, he would want a say. Thus, sharing decisions on time schedules or other logistics could still be important for the melanoma patients.

### 3.2 The ambivalence of making decisions

Variance in treatment options existed for patients with breast and prostate cancer. Making preference‐sensitive decisions for surgery or oncological treatment required individual involvement. Patients found this challenging. For instance, when the women with breast cancer were presented with treatment options that necessitated choice, such as whether they should undergo breast‐conserving surgery or have a mastectomy, they were pulled off the safe trail of the CPP. Insecurity and doubt about which decision to make followed. This represented another turning point in the patients' narratives—from leaning on professional decision‐making to actively participating in decisions. Many of the breast cancer patients considered themselves incompetent in making medical decisions. They looked for assurance that options were equal regarding the outcome and typically sought recommendations from their physician, as stated by two participants:

I think it is a medical decision whether you should keep your breast or not. So, I would do what is considered the safest option. (Participant 15).

I felt insecure when they wanted me to be part of the team; I mean, who am I to have an opinion on whether it is best to conserve the breast or not? (Participant 4).

Through their stories, the women imagined their tumour as an alien element that had invaded their bodies and disrupted their normal lives. This narrative analogy became part of how these women interpreted the standardization of the CPP. The standardization was seen to ensure choosing the best option to increase survival. However, seeing the tumour as an alien element also led some women to become reluctant to undergo breast‐conserving surgery, believing that a total mastectomy would be the safest option. One woman diagnosed with unilateral breast cancer considered having both breasts removed. However, wishes that did not correspond with medical guidelines were not supported by professionals. After expressing her fears and discussing treatment options, the final decision, although shared with a professional, put medical evidence above patient preferences. Hence, decision‐making was limited within the regime of standardization.

The men with prostate cancer had entered the CPP through prostate‐specific antigen (PSA) screening by their GP. As we saw with women with breast cancer, the cancer suspicion represented a turning point in the lives of these men. In their narratives, they immediately sought to control the situation. If they lacked information, they reported actively requesting it, perceiving it as one of their responsibilities as patients to obtain information necessary to handle the situation. Others had requested detailed and individually adapted knowledge when presented with preference‐sensitive treatment options. Thus, they sought professional recommendations to enable their decisions. Their requests for advice were not always met, which meant that the decisions were left to the patients. One of the participants narrated it in the following way:

I asked ‘What do you recommend?’ and he could not answer; they would not recommend one thing over the other […]. I think that is strange, coming from doctors who work in the field […]. I was surprised that they did not come up with a proper explanation of what was best. (Participant 15).

Even when patients positioned themselves as autonomous decision‐makers, sharing the decision with professionals could be necessary in the face of treatment options. In their stories, seeking advice from healthcare professionals became a way to individualize standards.

### 3.3 Opposing standardizations and pushing for action

Even though the patients were reluctant to participate in making medical decisions, they could have strong preferences in terms of logistics, such as being treated at a certain hospital. Their individual wishes sometimes strayed from the standardizations of the CPP. For instance, some patients found that their legal right to choose between hospitals was hindered by the structure of the CPP. Due to long travel distances, one woman requested to have her treatment in a different hospital to the one she was assigned. However, she experienced the right to be counteracted by professionals who sought to persuade her to comply with standardizations. In her narrative, she positioned herself as an individual who stood up for herself, fighting for her right to choose. This included rejecting professional recommendations and being willing to accept extended waiting times that were the consequence for patients straying from the CPP standards. This is her story:

When I called them to say I wanted to go to the central hospital, they told me ‘You can't, it is full, and we have much better capacity here’. But then I said, ‘That is not for you to decide. I decide that, and if you look at the map you understand why. I can't travel this long distance when I can reach the central hospital in just a couple of hours, and that is what is best for me’. […] So, they wouldn't accept it at first; they even called me and tried to convince me about the regional hospital. […] They told me that I might have to wait much longer if I kept up my choice. ‘That's a risk I am willing to take, so just send me there’, I said. (Participant 3).

In this story, the individual's personal needs came second to the hospital's need to meet the standards for waiting time within the CPP. A similar story about resistance was told by a woman who feared undergoing mammography screening as part of her diagnostic process. Rather than standardized mammography, she requested an ultrasound examination. In her experience, the healthcare system could not cope with this request. Her narrative consisted of multiple stories of having to explain and justify her choice at every encounter with healthcare professionals during her diagnostic process. Upon diagnosis, this participant subsequently conformed to professional requirements and underwent mammography as a final step before surgery.

The men with prostate cancer positioned themselves as individuals pushing for action to secure their treatment trajectory. PSA screening was not a standard offer, but these men had requested it from their GP. However, in accordance with medical guidelines, their GP typically suggested monitoring the PSA levels instead of transferring the men into the CPP as the levels increased. The men provided their narratives with a shift, illustrating how they themselves sought to ensure an active approach in which the tumour was surgically removed, rather than following a passive ‘wait and see’ approach by their GP.

Then levels started rising, but he [the GP] would still wait, but I said I wouldn't wait […] So he didn't act as I think he should have done, but I understand that it's not that easy for them either […] In the end, I just called him and asked to be referred. (Participant 16).

Taking on a responsible role, some of these men felt guilty about not being active enough, for instance, if postponing phone calls to request examinations. These stories showed how the men aimed to take control, but also how they potentially blamed themselves if they failed to control the situation.

When presented with treatment options, some men found that these options were limited due to advanced cancer disease. For instance, one of them had been diagnosed with an inoperable tumour and was offered radiation and hormone therapy instead of surgery. Others were provided suggestions of active monitoring or immediate radical treatment. Faced with these choices, the men expressed discomfort with monitoring and preferred radical surgery over radiation therapy, indicating that it was important for them to have the tumour physically removed.

He [the physician] presented me with two options: Either radiation or surgery. And then he presented the disadvantages with each of them, and I said, ‘I am having surgery’. (Participant 13).

Some men referred to themselves and the physicians as ‘we’, phrased in sentences such as ‘we agreed’. This way of voicing their experiences suggests that they saw the decision‐making process as a collaboration, indicating experiences of a patient–professional partnership in which decisions were shared.

## 4 DISCUSSION

The present study explored decision‐making experiences within different CPPs. Patients' experiences ranged from autonomous decision‐making to nonparticipation. Overall, standardizations were understood by patients as evidence‐based and patients trusted healthcare professionals to make decisions on their behalf. When facing preference‐sensitive treatment choices, patients sought recommendations from healthcare professionals. Patients with individual preferences outside CPP standardizations found that such preferences complicated shared decision‐making.

Treatment decisions have traditionally been the domain of the physician, potentially leading to paternalism.^8^ Encouraging patient participation in health services has provided patients with shared decision‐making on medical treatment.^8^, ^28^ However, patients in the current study associated CPPs with evidence‐based knowledge and trusted that they would benefit from a standardized trajectory. Previous research found that serious illness may enhance people's vulnerabilities and affect their autonomous capacity for decision‐making.^11^ Likewise, within emergencies, patients may not be willing or able to participate in making decisions.^13^, ^29^ For the patients in the current study, there were variations in whether the cancer diagnosis represented an emergency. For the breast cancer patients, experiencing emergency permeated their narratives and led them to embrace the standardized pathway over shared decision‐making. The narratives of the prostate cancer patients, on the contrary, contained experiences of health professionals suggesting monitoring the disease rather than immediate action. This indicates that patients' experience of emergency might influence their willingness to participate in shared decision‐making.

Previous research suggests that patients may prefer a more passive role in the initial stages of the CPP, but that their confidence in decision‐making often increases as their trajectory unfolds.^8^ When faced with preference‐sensitive treatment choices, patients in the present study sought medical recommendations and clear advice from healthcare professionals. Making decisions involves the potential of making the wrong decisions, which could be an additional stressor to individuals who are facing an existential crisis.^30^ As patients depend on healthcare professionals to restore their health,^11^ they may apply a ‘doctor knows best’ attitude and defer from participating to ensure good relationships with healthcare professionals. Some patients prefer professionals to make decisions, even those that are preference‐sensitive.^31^ Patient participation could include the patient remaining passive in certain contexts.^13^ A passive position could be an active choice, as shown by our participants. This could explain why patients in the current study who positioned themselves as autonomous decision‐makers expected professional advice on treatment options. Paradoxically, and as experienced by participants in the present study, healthcare professionals may be reluctant to recommend one specific solution for a preference‐sensitive choice out of respect for patient autonomy.^15^ This illustrates the complexity of patient participation in clinical practice.

In the present study, the predictability of the CPPs was appreciated by the patients. However, there are shortcomings in standardizations that do not respond to individual needs or wishes. Standardization could benefit the health service organization without being beneficial to patients.^5^ Our findings suggest that choice is encouraged when it is within the standardized system, but not when patients have preferences beyond standard solutions. As one of the policy goals of CPPs is to ensure patient participation and shared decision‐making, health services need to consider whether patient participation should be implemented within the CPP or if each patient should fit into a standardized logistics. It is important that standardization is used as a tool for designing good services, not as an aim for the service.

## 5 LIMITATIONS

This is one of only a few studies examining patient experiences with CPPs in Norway, and the only one exploring shared decision‐making within these CPPs. The study design consisted of retrospective qualitative interviews with cancer patients. This design has some weaknesses for studying shared decision‐making. First, the interviews covered general patient experiences with CPPs and might not have assessed all sides of shared decision‐making, since this was only one of several topics in the interviews. Moreover, a different design including observations of meetings between health professionals and patients, or interviews with health professionals, could have provided relevant information that is missing within our study design.

The current study presents patient narratives from three CPPs and may not be transferable to patients' experiences with other CPPs. The stories were not equally distributed with respect to volume, which could have influenced our findings. However, we consider the individual narratives important for developing knowledge about shared decision‐making within CPPs. The low number of patients recruited with prostate cancer and malignant melanoma diagnoses suggests that the individuals who decided to participate had a specific interest in telling their stories. As recruitment of participants from these two groups ended due to availability, saturation within each group of cancer patients is a limitation of the study. Wanting to be prepared for their interview, some participants read about CPPs before attending research interviews. This could have influenced how they responded during the interviews. The interview guide did not have a narrative approach, which might be another limitation of the study. Participants, however, narrated their stories freely during the interviews.

## 6 CONCLUSION

Narratives from different cancer pathways suggest varied opportunities for shared decision‐making. Opportunities were experienced when standard action towards the specific cancer diagnosis allowed preference‐sensitive treatment options. A standardized patient pathway also meant predictability and safety for patients during their cancer trajectory. However, standardizations could lead professionals to overlook individual needs. Balancing standardization towards individualized care is necessary to facilitate patient participation in CPPs. A discussion on what patient‐centred care implies within each of the standardized CPPs is warranted among health policy makers, service providers and patient organizations.

## CONFLICT OF INTERESTS

The authors declare that there are no conflict of interests.

## AUTHOR CONTRIBUTIONS

Marit Solbjør, Tone Andersen‐Hollekim and Line Melby designed the study. Marit Solbjør and Kari Sand collected the data. All authors contributed to the analysis and interpretation of data. Tone Andersen‐Hollekim and Marit Solbjør drafted the manuscript. All authors have contributed to revising this article critically and contributing with important intellectual content. All authors have given final approval of the version to be published and take public responsibility for the content. All authors agree to be accountable for all aspects of the work.

## Data Availability

The data that support the findings of this study are available on request from the corresponding author. The data are not publicly available due to privacy or ethical restrictions.
